# Synergistic Effect of Zinc-Chitosan Nanoparticles and Hydroxychloroquine to Inhibit Buffalo Coronavirus

**DOI:** 10.3390/polym15132949

**Published:** 2023-07-05

**Authors:** Anju Manuja, Balvinder Kumar, Dharvi Chhabra, Basanti Brar, Riyesh Thachamvally, Yash Pal, Minakshi Prasad

**Affiliations:** 1 ICAR-National Research Centre on Equines, Hisar 125001, India; 2 Lala Lajpat Rainiversity of Veterinary & Animal Sciences, Hisar 125004, India

**Keywords:** coronavirus, bovine, chitosan, ZnO, nanoparticles, hydroxychloroquine

## Abstract

Zinc ions can hinder the synthesis of proteins required for accomplishing several stages of the viral life cycle. The intracellular zinc concentration can be increased by using zinc ionophores which transport zinc ions into the cells and hinder viral replication. (Hydroxy)chloroquine is an example of a zinc ionophore, but both zinc and (hydroxy)chloroquine can be toxic to the host organism. The nanocarriers may serve as camouflage to evade the adverse effects of drugs, chemicals, and nanoparticles on the host. We formulated ZnO nanoparticles with flower-like morphology (ZnONFs). It was further decorated with chitosan along with hydroxychloroquine (as a zinc ionophore) (CHCZnO NPs). We have chosen the cationic polymer chitosan since it is biocompatible, biodegradable and binds easily with the cells, and enhances the transport of drugs across cell membranes. The formulation was investigated for size, shape, surface charge, and interaction of chemicals used. We evaluated the formulations for cytotoxicity, and biocompatibility in embryonated chicks and their efficacy against bovine coronavirus (BCoV) isolated from a buffalo calf, and pneumo-enteric coronaviruses isolated from a buffalo calf with promising results in comparison to ZnONFs/hydroxychloroquine alone. Furthermore, we elucidate the mechanism underlying the lysosomotropic effect of various formulations on Vero cells infected with the buffalo coronavirus.

## 1. Introduction

Zinc ions play a vital role in several activities of “cellular enzymes” and “transcription factors”. Zn^2+^ is possibly a crucial co-factor for several viral proteins. An increase in intracellular zinc levels can impede the activity of RNA-dependent RNA polymerases, which are essential for the synthesis of proteins required for various stages of the viral life cycle. The intracellular zinc concentration can be increased by using zinc ionophores which facilitate the zinc ions into the cells and subsequently impair the virus replication. Zinc also exhibits anti-inflammatory properties by hampering NF κB signaling and modulation of regulatory T cell functions that may reduce the cytokine storm. The dispersion of ZnONPs in an aqueous medium such as water is difficult. We developed dispersible zinc oxide nanoparticles resembling flower-like structures (ZnONFs) using a unique technique in a short time while offering a high quantity of ZnONFs.

Coronaviruses are a family of viruses that infect mammals and birds, causing a range of respiratory illnesses ranging from mild to severe. They are enveloped viruses with a single strand of positive-sense RNA. In humans, coronaviruses can cause respiratory infections as observed in SARS, MERS, and the global pandemic COVID-19 infection. Most coronaviruses are found in domestic as well as wild animals, and it is possible that they jumped to the human population through animals. Bovine coronavirus (BCoV) is one of the coronaviruses of veterinary significance causing pneumoenteritis in bovines [1].

Chloroquine phosphate, an aminoquinoline medication that was previously used to treat malaria, has been shown to act as a zinc ionophore [2]. It has also been shown to have noticeable efficacy against pneumonic symptoms in COVID-19 patients in clinical trials performed in China [3]. Hydroxychloroquine (HC) is a lysosomotropic autophagy inhibitor that can be used either unaccompanied or accompanied by chemotherapy [4]. Chloroquine phosphate has a weak base that increases the endosomal pH, is quickly absorbed into acidic vesicles, and disrupts terminal glycosylation, preventing the virus from surviving. Chloroquine inhibits human immunodeficiency virus (HIV) [5], influenza virus [6], dengue virus (DENV) [7], Japanese encephalitis virus (JEV) [8,9], West Nile virus (WNV), and Zika virus [10] infection by inhibiting pH-dependent phases of viral replication. Zinc and chloroquine are both FDA-approved and widely available. Chloroquine targets zinc in the lysosomes, and when given along with zinc, it intensifies the cytotoxicity and induced apoptosis [2].

The most extensively recognized (hydroxy)chloroquine adverse effects are gastrointestinal side effects, hepatotoxicity, and/or blindness. Combining anti-flu medicines such as lopinavir/ritonavir with chloroquine or hydroxychloroquine could cause severe arrhythmias and drug interactions [11]. The long half-life and the enormous volume of appropriation of the two medications can identify these toxic levels.

Given the suggested antiviral action of zinc ions, chloroquine, and their contribution to lysosomal processes, we aimed to offer an alternative for ameliorating the adverse effects of chloroquine alone as an interferon blocker and its action as a zinc ionophore in combination with zinc compounds [12]. We focused on delivery applications using chitosan as a functional carrier for zinc and chloroquine to reduce the adverse effects of both components. The nanocarriers serve as camouflage to avoid any harmful effects of the drugs, chemicals, or nanoparticles on the host. We have chosen a polysaccharide, cationic polymer chitosan since it is biocompatible, binds easily to the drug, and is biodegradable. It also helps transport drugs across cell membranes.

The formulations were investigated for size, shape, surface charge, and interaction of various chemicals used during synthesis. We also evaluated the formulations for cytotoxicity and their efficacy against the buffalo coronavirus. Furthermore, we elucidate the mechanism underlying the lysosomotropic effect of various formulations on Vero cells infected with buffalo coronavirus.

## 2. Materials and Methods

### 2.1. Materials

Chitosan (a deacetylated form of chitin with 75–85% deacetylation) was obtained from Sigma-Aldrich Chemicals Private Ltd. in Bangalore, India. Zinc sulfate was procured from Qualigens Fine Chemicals Pvt. Ltd. in Mumbai, India, while hydroxychloroquine was purchased from Ipca laboratories in India. Life Technologies Co. in Carlsbad, CA supplied FluoZin-3 AM and LysoTracker probes for the study. Other analytical-grade chemicals and reagents were obtained from Sigma-Aldrich Chemicals Private Ltd. The cell lines used in the study were obtained from the National Centre for Veterinary Type Cultures in Hisar, India, and were cultured in a specific media containing fetal bovine serum, antibiotics, and other ingredients. The cells were incubated at a specific temperature and humidity in a CO_2_-rich environment. The glassware used in the study was from M/S Borosil in India.

### 2.2. Synthesis of Hydroxychloroquine/zinc Oxide Nanoparticles Loaded Chitosan Composites

We synthesized dispersible ZnO nanoparticles showing morphology in the shape of flowers (ZnONFs) via a pulse on and off using a microwave-assisted rapid method as described previously using zinc sulfate as a precursor as described previously [13]. Chitosan 1% was prepared in acetic acid *w*/*v*, chitosan (0.1–0.01%) in DW *v*/*v*, hydroxychloroquine (0.0012 to 0.012%), and ZnO solution (0.05–0.75%) were prepared, mixed thoroughly and passed through a 0.22 µM filter membrane and refrigerated at 4 °C.

### 2.3. Morphology, Particle Size, and Surface Charge Determination (ζ)

With the “Zetasizer nano ZS” Malvern Instruments, Malvern, UK, the mean particle size and its distribution (polydispersity index) of synthesized ZnONFs and hydroxychloroquine/zinc oxide nanoparticles loaded chitosan composites (CHCZnO NPs) were measured at 250 °C using “dynamic light scattering”. The laser light scattering method was used to investigate the zeta potentials of the identical samples. It can also predict colloid dispersion stability.

The morphology/shape of the ZnONFs and CHCZnO NPs was observed through a scanning electron microscope (Hitachi S-3400N’) at 13 kV and 80 KV and by a transmissible electron microscopy. The samples for SEM were placed onto metal stubs with adhesive tapes. EDAX detector (AMETEK, Berwyn, PA, USA) was utilized for element detection in samples in the current investigation.

### 2.4. Estimation of Zinc Concentration in Formulations

The quantities of zinc in ZnONFs and CHCZnO NPs were determined by an atomic absorption spectrophotometer (PerkinElmer, Norwalk, CT, USA) as mentioned previously [13,14].

### 2.5. Fourier Transform Infrared Spectroscopy (FTIR)

We used an IR spectrophotometer (Perkin Elmer Spectrum BX II, Waltham, MA, USA) to analyze the samples. The interaction of several chemicals taken during synthesis was investigated using “Fourier Transform Infrared Spectroscopy” of ZnONFs, HC, and CHCZnO NPs.

### 2.6. Cytotoxicity of Nanoformulations

In this study, the cytotoxicity of ZnONFs, CHCZnO NPs, and HC was evaluated on African monkey kidney (Vero) cells grown in a specific medium as previously described [15]. The cells were seeded in a 96-well cell culture plate and allowed to grow until they reached approximately 70% confluency. ZnONFs, CHCZnO NPs, and HC were added to the cells at various concentrations ranging from 2000 mg/mL to 62.5 mg/mL @10 µL per 100 µL media and incubated for one day. The next day, a resazurin dye was added to all the wells, and the optical densities were measured using a Biotek Instruments Powerwave X2 after 4 h. The cytotoxicity was calculated based on the untreated cells and the background coloration of the media, and the inhibitory concentration (IC_50_) was calculated using a specific equation [16].

Y = Min + (Max − Min)/1 + (X/1C_50_) n_H_ (1)

where Y is the response value along with maximum (Max) and minimal (Min) values, and X is the inhibitory concentration. n_H_ is Hill coefficient.

### 2.7. Biocompatibility in Embryonated Chicken Eggs

Biocompatibility of our formulation CHCZnO NPs was confirmed in specific pathogen-free embryonated eggs on day 10. We inoculated them with two different doses (containing 1.87 and 0.94 mg/mL of ZnO NFs); based on cell culture toxicity studies) or phosphate-buffered saline as a control, in duplicates, in a 50 µL quantity/per 50 gm egg in the allantoic cavity. To test the biocompatibility of the formulations in chick embryos, eggs were examined for viability up to five days after inoculation.

### 2.8. Virus Propagation in African Green Monkey Kidney Cells (Vero Cells)

Buffalo coronavirus strain, (BCoV) isolated from a diarrheic buffalo calf from Haryana, India, (kindly provided by Dr. Minakshi Prasad LUVAS, Hisar, India) was inoculated in Vero cells. Briefly, Vero cells were infected with BCoV and incubated at 37 °C. After one hour, the inoculum was replaced with growth medium containing fetal bovine serum (FBS) and antibiotics. The conditioned medium was then harvested after 4 to 6 days, centrifuged to remove cells and debris, and sterile-filtered. The Vero cells in triplicates were then infected with BCoV for one hour and the cell lysates were prepared by a rapid freeze–thaw method and stored at −80 °C.

### 2.9. RT-PCR

RNA was extracted from virus-infected, subsequently treated, and uninfectedcells using Trizol (Sigma, Aldrich, Burlington, MA, USA), as per the manufacturer’s instruction. Extracted RNA from each sample was further transcribed using specific primers of the M gene with “MMLV reverse transcriptase enzyme” using an I Script cDNA Synthesis kit (Biorad, Hercules, CA, USA). We used polymerase chain reaction to obtain amplicons of the BCoV M gene from complementary DNA, using the following amplification conditions: initial denaturation at 94 °C for 5 min, 35 cycles of denaturation at 94 °C for 30 s, annealing at 55 °C for 30 s, extension at 72 °C for 30 s, and final extension at 72 °C for 7 min. The fragments of 523 bp were observed on 1.5% agarose gel and were sequenced after purification.

### 2.10. Effects of Formulations on African Green Monkey Kidney Cells (Vero Cells)

Vero cells, in triplicates, were infected with BCoV for one hour at 37 °C and the cells were then washed thrice with PBS. The virus-infected Vero cells were treated with ZnONFs, CHCZnO NPs, and HC, at different dilutions. We used untreated and uninfected cells as a negative control, whereas virus-infected cells without treatment were kept as a positive control. The cell morphologies and viral cytopathic effects in treated cells and untreated uninfected controls were observed.

Further, the ability of formulations to inhibit the virus before infecting the Vero cells was explored. Vero cells were exposed to BCoV for 1 h, and then media was replaced and treated with ZnONFs, and CHCZnO NPs at dilutions 1:4; 1:8, and 1:16 (0.187, 0.094, and 0.047 mg/mL of ZnO NPs, respectively), @10 µL/100 µL of media. After 48 and 72 h, the effect of treatment was observed on the morphology, proliferation, and CPE. Cell proliferation was determined visually as well as by the calorimetric method using resazurin dye after normalizing with the background.

### 2.11. Real-Time Quantitative PCR

We quantified the BCoV in Verocells using real-time quantitative PCR (qPCR). We transcribed extracted RNA from each sample using random hexamer primers, according to the protocol provided by the manufacturer (Biorad, USA). We ran qPCR using a 10 µL SYBR Green Supermix (qPCR Mastermix) and 10 nM N gene-specific primers of BCoV (forward primer: 5′-TGGATCAAGATTAGAGTTGGC-3′, reverse primer: 5′-CCTTGTCCATTCTTCTGACC-3′) on a thermal cycler QuantStudio™ 3 (Thermo Fisher Scientific, Waltham, MA, USA) with the following conditions: one cycle of initial denaturation at 95 °C for 5 min, 40 cycles of 15 s denaturation at 95 °C, 60 s annealing at 60 °C. We determined the threshold cycles (Ct) and calculated the relative fold change in the N gene of BCoV using the 2^−ΔΔCt^ method.

### 2.12. Intra-Cellular Zinc Uptake

In this study, the presence of lysosomes and intracellular free zinc ions were detected using LysoTracker and FluoZin3 probes under a fluorescence microscope. Vero cells were seeded in a 12-well plate and treated with ZnONFs, CHCZnO NPs, and HC at various concentrations for 30 min. The cells were then incubated with a mixture of LysoTracker and FluoZin3 for an additional 30 min, after which the medium was removed, and the cells were washed with HBSS. The cells were then observed under a fluorescent microscope and the colocalization of zinc ions and lysosomes was determined by merging the green and red images. The fluorescence intensity was quantified using NISElements AR software from Nikon Instruments.

Zinc ions were revealed as green with an excited wavelength of 490/20 nm and an emission wavelength of 528/38 nm. Lysosomes were detected as red with an excited wavelength of 555/28 nm and an emission wavelength of 617/73 nm.

### 2.13. Statistical Analysis

We used the mean and standard deviation of at least three independent experiments to represent the data and compared differences between experimental groups using GraphPad software [17] with a student’s *t*-test. At *p* < 0.05, substantial differences were evaluated.

## 3. Results and Discussion

### 3.1. Characterization/Description

#### 3.1.1. Size, Morphology, and Charge

**Figure 1:**
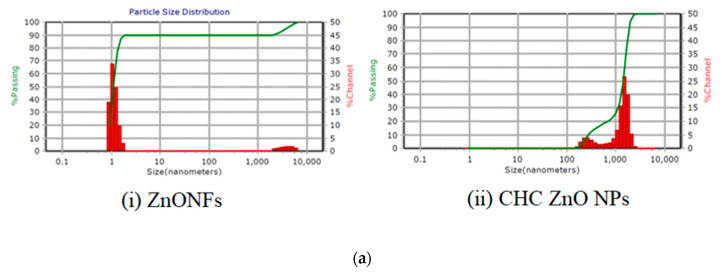

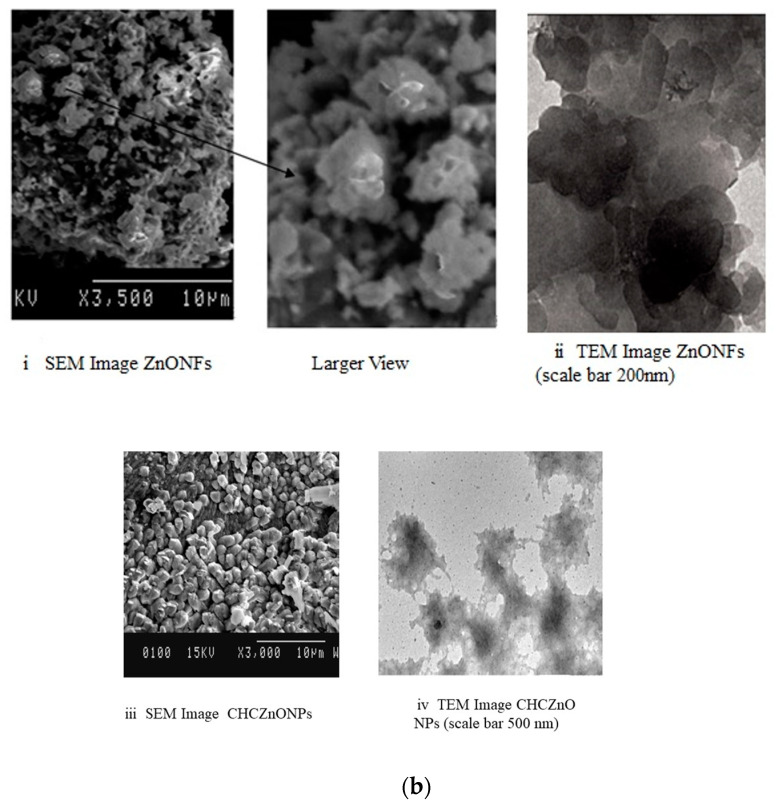
(**a**) Particle size analysis of (**i**) ZnONFs; (**ii**) CHCZnO NPs. (**b**) Images of zinc oxide nanoparticles (ZnONFs) and CHCZnO NPs. (**i**) Scanning electron microscope (SEM) image of ZnONFs. (**ii**) Transmission electron microscope (TEM) image of ZnONFs. (**iii**) SEM image of CHCZnO NPs. (iv) TEM image of CHCZnO NPs.

The average size of the particles (diameter) observed by the zeta sizer was less than 10 nm with a 0.0 polydispersity index (PDI) indicating mono dispersion of the ZnONFs (Figure 1a(i)). The particles of CHCZnO NPs (Figure 1a(ii)) were in the range of 252.9 to 1469 nm with PDI 0.282 and viscosity 0.852. Light scattering techniques, which measure volume distribution, are particularly useful for large particles. Chitosan nanoparticles have a high swelling capacity, so the hydrodynamic diameter measured by dynamic light scattering is larger than the size measured by electron microscopy. As a result, detecting a few bigger particles, originating from particle aggregation is extremely sensitive.

The zinc oxide nanoparticles were observed as assorted petals in the shape of flowers as determined by (i) SEM and (ii) TEM (Figure 1b). CHCZnO NPs appeared as individual particles in SEM (iii) and TEM (iv)

The stability of nanoparticle dispersions can also be predicted using the zeta potential. The zeta potential of ZnONFs and CHCZnO NPs was 200 mV. Zeta potential provides a prediction about the stability of nanoparticle dispersions. A high zeta potential >|30|mV can provide an electric repulsion, preventing particles from clumping together. Increases in zeta potential are accompanied by increases in particle surface charge. The zeta potential (200 mV) is sufficient for forming a stable nanoparticle suspension. As the zeta potential grows, the repulsive interactions between the particles become bigger, resulting in the creation of a more stable suspension with a more uniform size distribution. ZnONFs showed negative polarity, whereas the polarity of CHCZnO NPs remained undetermined. It may be due to a combination of cationic chitosan and anionic ZnONFs.

**Figure 2:**
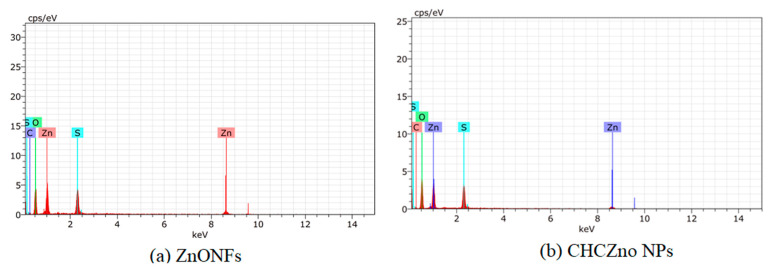
Energy dispersive X-ray spectra (EDS) of (**a**) ZnONFs and (**b**) CHCZnO NPs with elemental quantification.

Figure 2 shows the “energy-dispersive X-ray” spectra (EDS) of ZnONFs and CHCZnO NPs, along with elemental quantification. Zinc was estimated as 43.00% and 33.79% in ZnONFs and, CHCZnO NPs, respectively, whereas oxygen “O” was observed higher in CHCZnO NPs, followed by ZnONFs. We also observed sulfur “S” in ZnONFs and CHCZnO NPs as the zinc sulfate was employed in the fabrication of NPs (Table 1).

#### 3.1.2. Fourier Transform Infrared Spectroscopy

**Figure 3:**
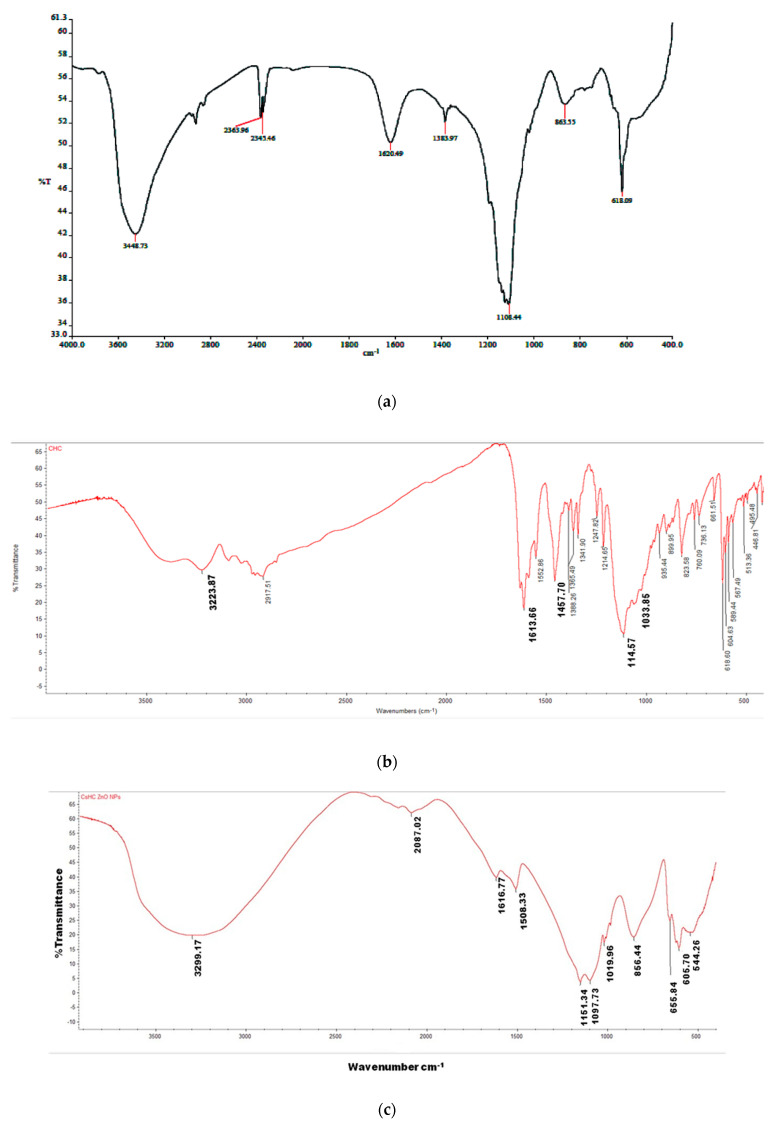
Fourier transform infrared spectroscopy (FT-IR) of (**a**) ZnONFs (**b**) HC (**c**) CHCZnO NPs.

The connections of various chemicals employed during fabrication were investigated. We used Fourier transform infrared spectroscopy (FT-IR) to analyze the ZnONFs and CHCZnO NPs. Figure 3 shows the FT-IR spectra of the ZnONFs, hydroxychloroquine, and CHCZnO NPs. The FT-IR spectrum of the ZnONFs was collected in the 400–4000 cm^−1^ range. The stretching vibration of the -OH bond is shown by the peaks between 3422 cm^−1^ and 3448 cm^−1^. The initial overtone of the basic stretching mode of –OH is given to the peaks from 1618–1624 cm^−1^. The band at 618 cm^−1^ is due to the Zn–O stretching mode. At 2917.51 cm^−1^, the “aromatic C-H stretching” of HC was observed. The –OH stretching frequency appeared at 3223.87 cm^−1^ of hydroxychloroquine is moved to 3299.17 cm^−1^ in the CHCZnO NPs. The “aromatic ‘C=C” extension in regions 1613.06 and 1457.70 cm^−1^ in HC is adjusted to 1616.77 and 1508.33 cm^−1^, respectively, in CHCZnO NPs. Likewise, the “C-Cl” extensive/stretching frequency at 1033.85 cm^−1^ too displayed a remarkable displacement to 1019.96 cm^−1^ in the inclusion compound. The “C-N” “bending frequency” at 1114.57 cm^−1^ in the HC is shifted to 1157.29 cm^−1^ in CHCZnO NPs. Both molecules are present in the formulation, as evidenced by the significant alterations in the FT-IR spectra of ZnO NFs/HC/CHCZnO NPs.

### 3.2. Cytotoxicity Studies on African Green Monkey Kidney Cells (Vero Cells)

**Figure 4:**
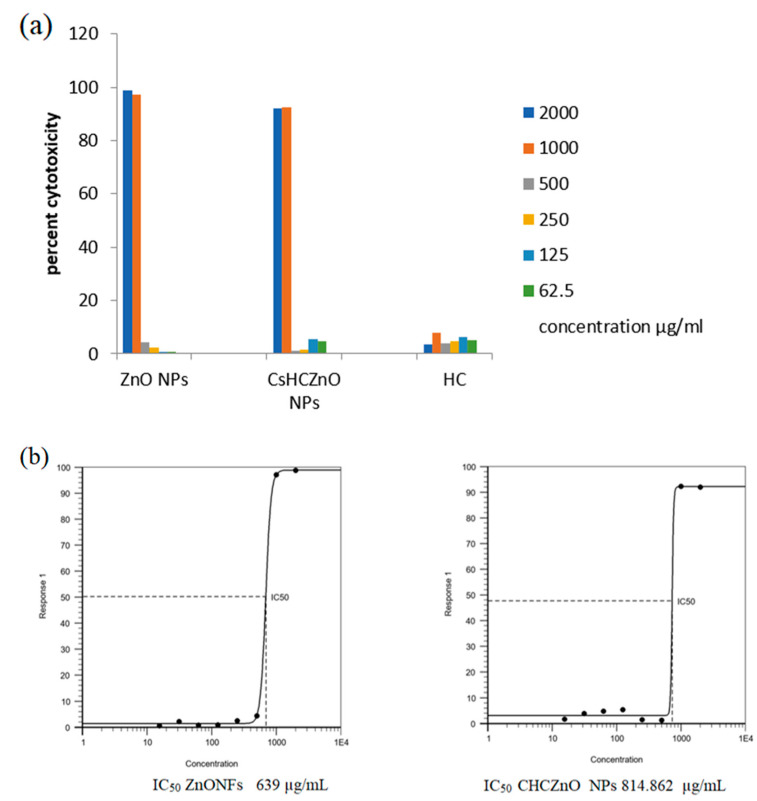
(**a**) The cytotoxicity of zinc oxide nanoflowers (ZnONFs), CHCZnO nanoparticles (NPs), and hydrochloroquine (HC) was tested on Vero cells. The metabolic activity of the cells was measured by a resazurin assay after 24 h of incubation. (**b**) The cytotoxic percentage and the half-maximal inhibitory concentration (IC_50_) were determined for the ZnONFs and CHCZnO NPs.

When exposed to a biological environment, metal nanoparticles tend to disperse and release ions [18]. The cytotoxic activity of ZnONFs, CHCZnO NPs, and HC was investigated by a colorimetric analysis using resazurin. The results indicated that ZnONFs were more toxic than CHCZnO NPs at concentrations of more than 1000 µg/mL (Figure 4a,b). The differences in harmful effects at higher concentrations were found to be significant statistically (10 µg/mL and 5 µg/mL, *p* < 0.05). At low concentrations, we observed a meager rate of cytotoxicity. The mechanism regarding the toxicity of ZnONFs was principally based on the formation of “reactive oxygen species” [18]. These data were plotted, and Hill 4 parameter sigmoidal regression was performed on AAT BIoquest. IC_50_ calculated for ZnONFs was 639 µg/mL and for CHCZnO NPs was observed as 814.86 µg/mL.

### 3.3. Biocompatibility in SPF Chick Embryos

**Figure 5:**
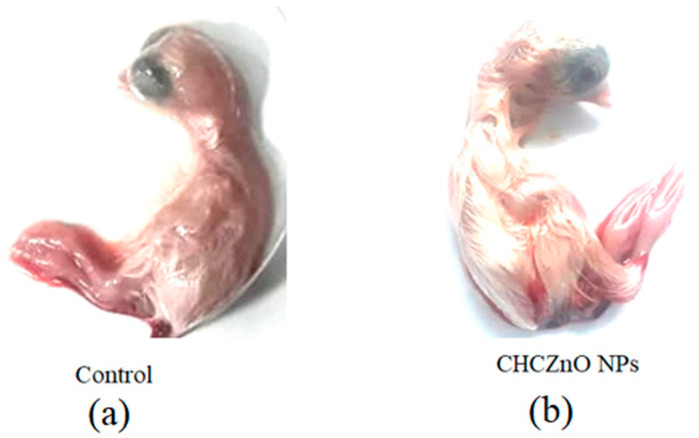
Biocompatibility in SPF chick embryos (**a**) PBS (control). (**b**) CHCZnO NPs.

The early stages of chickens’ rapid embryonic growth provide a sensitive model for investigating toxicity. Three identical specific pathogen-free (SPF) embryonated chicken eggs were infected in triplicate with two different CHCZnO NPs dilutions (1:4 and 1:8) in 100 μL quantity through the allantoic route. The eggs were visualized daily for the first five days after injection to check for embryo mortality. None of the embryos showed any sign of toxicity suggesting the biocompatibility of synthesized CHCZnO NPs formulation. The representative figures of control, as well as CHCZnO NPs, treated embryos are shown in Figure 5.

### 3.4. Effects of Formulations on African Green Monkey Kidney Cells (Vero Cells)

**Figure 6:**
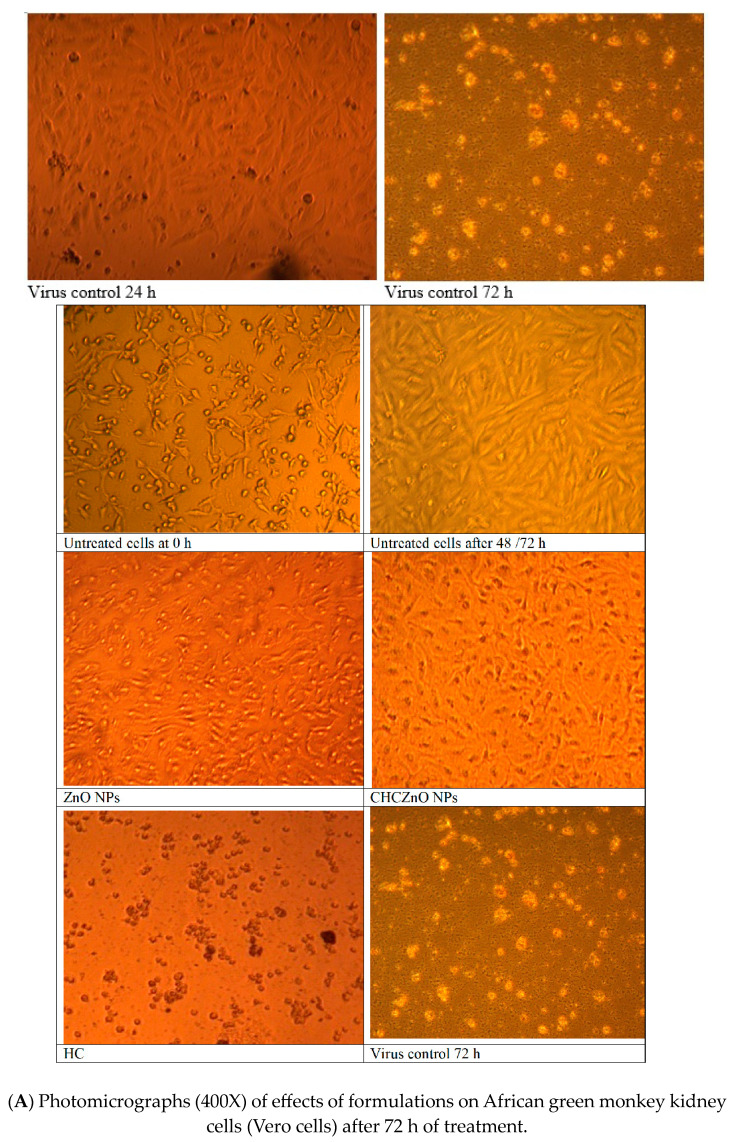

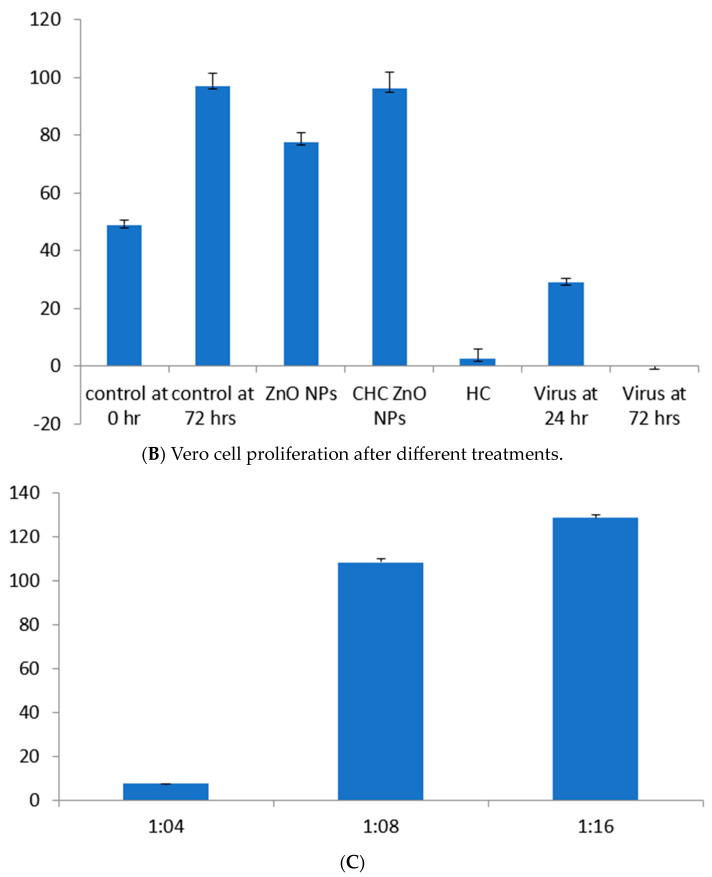
Influence of ZnONFs, CHCZnO NPs, HC on BCoV infectedVero cells. (**A**) Photomicrographs (400×). (**B**) Percent cell confluency. (**C**) Inhibitory action of different dilutions of CHCZnO NPs on virus infecting the Vero cells.

Vero cells, in triplicates, were infected with BCoV for one hour at 37 °C and the cells were then washed thrice with PBS. The virus-infected Vero cells were treated with ZnONFs, CHCZnO NPs, and HC, at different dilutions; untreated and uninfected cells were kept as a negative control, whereas virus-infected cells without treatment were kept as a positive control. The cell morphology of formulation CHCZnO NPs treated cells and untreated uninfected controls were in good conformity and showed better proliferation and monolayer formation with confluency of 96–100% followed by ZnONFs (77.5–80%). The representative figures are shown in Figure 6a. Cells treated with HC were deformed, aggregated, and degenerated depicting some morphological/detrimental effects on the cells. Virus control after 24 h started showing cytopathic effects (CPE) such as the rounding of cells and after 72 h of post-treatment rounding and desquamation of cells was visible. We did not observe CPE in the cells treated with any of the formulations as well as untreated ones. Figure 6b depicts the Vero cell proliferation after different treatments.

We further explored whether our formulations could inhibit the virus before infecting the Vero cells. We treated the virus with different concentrations of formulations and observed the infectivity in the Vero cells. Cell proliferation was determined visually as well as by the calorimetric method using resazurin dye after normalizing with the background. We could not observe good proliferation at 1:4 dilution of CHCZnO NPs, since it was toxic to the cells along with the virus (Figure 6c). However, 1:8 and 1:16 dilutions exhibited good cell proliferation even better than the controls suggesting chitosan-loaded ZnONFs promote the growth of the Vero cells. CHCZnO NPs contain 0.75% of ZnO NPs.

### 3.5. Buffalo Coronavirus-Infected Vero Cells Exhibited M Protein

We intended to determine the adaptation of BCoV on Vero cells. We extracted the RNA from BCoV-infected cells treated with different formulations as well as untreated control, transcribed by reverse transcription, and amplified M protein as per the details given earlier in the methodology section. The most abundant M viral protein is responsible for providing a specific form [19], attaching to the nucleocapsid, and managing coronavirus assembly [20]. Although coronavirus M-proteins from various genera have a wide range of amino acid compositions, they nonetheless have certain structural commonalities. The short amino terminus on the outside of the virion and the lengthy carboxy terminus on the interior of the virion are both located next to three transmembrane domains in the M-proteins [21]. In general, the viral scaffolding is maintained/sustained by MM interactions. In particular, the SARS-CoV 2 M-protein does not show amino acid substitutions in comparison to SARS-CoV [22].

**Figure 7:**
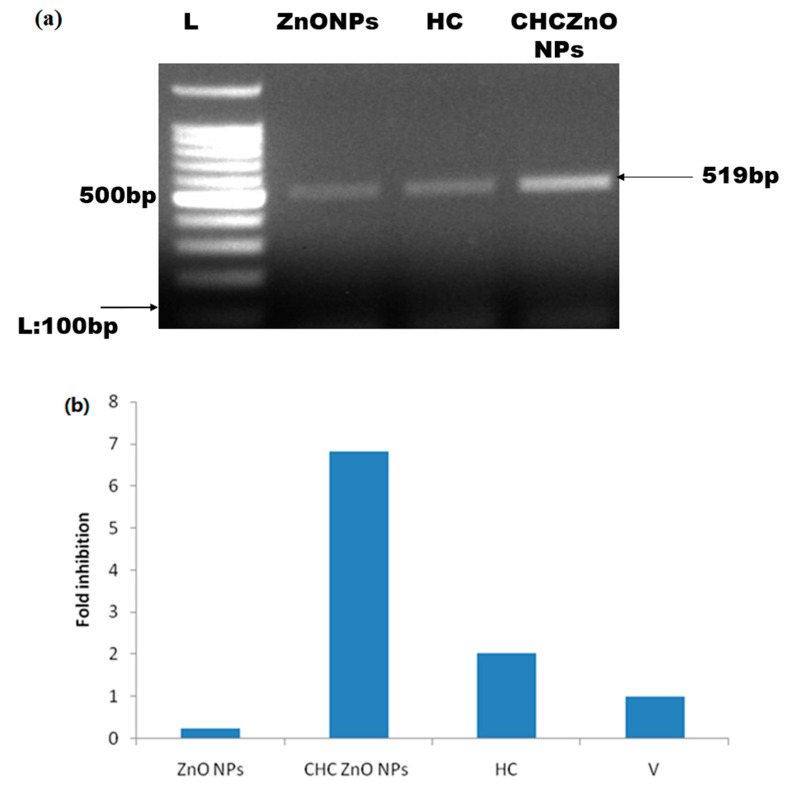
(**a**) Gel electrophoresis of PCR products of M gene of Buffalo coronavirus. RNA was extracted from the BCoV-infected Vero cells and transcribed. Amplicons yield 519 bp products of BCoV M protein in all the infected cells. (**b**) Relative fold change in N gene of BCoV in infected and treated Vero cells.

The positive control (only virus-infected samples) yielded 519 bp product specific to M-protein (Figure 7a). We could not observe the amplification in the negative control (uninfected and untreated samples). The representative samples were sent for sequencing to further confirm the buffalo coronavirus-specific M protein and submitted to GenBank NCBI and assigned a GenBank accession number MW592691 for this sequence. Using blast analysis, we observe 100% identity with the bovine coronavirus sequences in the database).

Further, we quantitated the levels of BCoV in the Vero-infected cells by quantitative real-time PCR to determine the relative fold change in the N gene of BCoV. CHCZnO NPs were able to inhibit 6.8-fold times the N gene as compared to controls, whereas HC could inhibit only 2 times (Figure 7b).

### 3.6. Nanoparticle Uptake in Vero Cells

**Figure 8:**
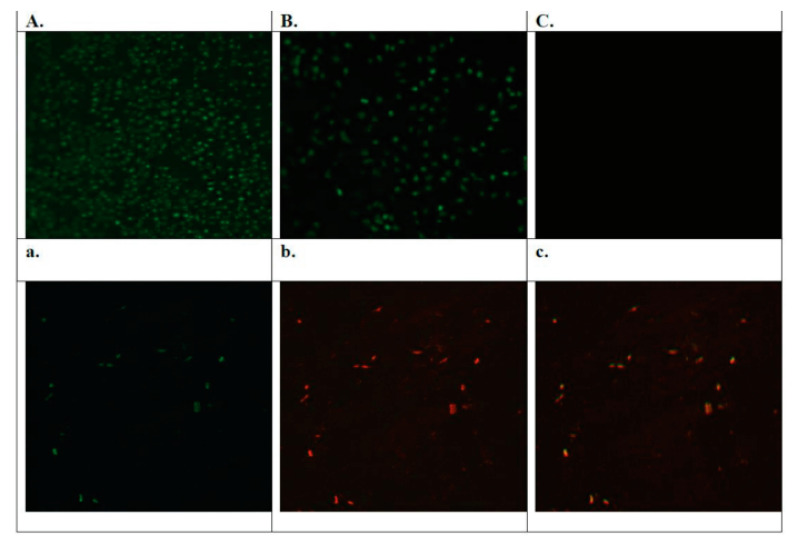
African monkey kidney cells (Vero cells) in a 96-well plate were treated with (**A**) ZnONFs, (**B**) CHCZnO NPs (**C**) hydroxychloroquine alone. In the lower panel shown are the representative images of stained polymeric composite CHCZnO NPs, with (**a**) FluoZin (**b**) lysomotracker (**c**) merged overlay. Under a fluorescent microscope, zinc ions were revealed as green with an excited wavelength of 490/20 nm and an emission wavelength of 528/38 nm. Lysosomes were detected as red with an excited wavelength of 555/28 nm and an emission wavelength of 617/73 nm.

Chloroquine is a well-known lysosomal targeting agent [23,24,25]. We investigated the detection of zinc distribution intracellularly after the treatment of Vero cells with ZnONFs alone, HC alone, and CHCZnO NPs. As shown in Figure 8, our ZnONFs and CHCZnO NPs treated cells showed zinc ions were mainly localized intracellularly in the lysosomes, as indicated by the colocalization of FluoZin-3/LysoTracker fluorescent signals. It indicates that our novel preparation of dispersible zinc oxide exhibits a lysosome tropic effect with or without chloroquine. Vero cells also uptake zinc ions from CHCZnO NPs which contain only 0.75% of ZnONFs. As reported earlier chitosan being a cationic polymer, also facilitates internalization. We did not observe any fluorescence or zinc internalization in HC-treated cells and controls. It indicates these formulations do not exhibit internalization or lysosome tropic effect, indicating some other factors facilitating cell proliferation and antiviral activity which need to be explored in further studies.

## 4. Conclusions

We provided a delivery vehicle or alternative in the form of CHCZnO NPs reducing the adverse effects of zinc and hydroxychloroquine. ZnONFs obtained in flower-like structures showed cellular internalization of zinc ions and demonstrated better synergy of chitosan, zinc oxide nanoparticles, and hydroxychloroquine to inhibit the bovine coronavirus. The polysaccharide cationic polymer chitosan attaches quickly to the negatively charged cell membrane, and along with hydroxychloroquine, it mediates the transport of zinc inside the cells. In conclusion, since both hydroxychloroquine and zinc are well thought out perspective components for the management of viral infections, the efficient delivery of amalgamation of these components may be a new stratagem for developing a more effective approach against coronaviruses.

## Figures and Tables

**Table 1:** Elemental quantification of ZnONFs and CHCZnO NPs.

Element	ZnONFs	CHCZnO NPs
Oxygen	35.56	43.55
Zinc	43.00	33.79
Carbon	6.48	6.98
Sulfur	14.96	15.68

## Data Availability

The data are available in the manuscript itself.

## References

[1] Weiss S.R., Navas-Martin S. (2005). Coronavirus pathogenesis and the emerging pathogen severe acute respiratory syndrome coronavirus. Microbiol. Mol. Biol. Rev..

[2] Xue J., Moyer A., Peng B., Wu J., Hannafon B.N., Ding W.Q. (2014). Chloroquine is a zinc ionophore. PLoS ONE.

[3] Gao J., Tian Z., Yang X. (2020). Breakthrough: Chloroquine phosphate has shown apparent efficacy in treatment of COVID-19 associated pneumonia in clinical studies. Biosci. Trends.

[4] Collins K.P., Jackson K.M., Gustafson D.L. (2018). Hydroxychloroquine: A physiologically-based pharmacokinetic model in the context of cancer-related autophagy modulation. J. Pharmacol. Exp. Therap..

[5] Tsai W.P., Nara P.L., Kung H.F., Oroszlan S. (1990). Inhibition of human immunodeficiency virus infectivity by chloroquine. AIDS Res. Hum. Retrovir..

[6] Ooi E.E., Chew J.S.W., Loh J.P., Chua R.C.S. (2006). In vitro inhibition of human influenza A virus replication by chloroquine. Virol. J..

[7] Farias K.J.S., Machado P.R.L., da Fonseca B.A.L. (2013). Chloroquine inhibits dengue virus type 2 replication in Vero cells but not in C6/36 cells. Sci. World J..

[8] Zhu Y., Xu Q., Wu D., Ren H., Zhao P., Lao W., Wang Y., Tao Q., Qian X., Wei Y.-H. (2012). Japanese encephalitis virus enters rat neuroblastoma cells via a pH-dependent, dynamin and caveola-mediated endocytosis pathway. J. Virol..

[9] Boonyasuppayakorn S., Reichert E.D., Manzano M., Nagarajan K., Padmanabhan R. (2014). Amodiaquine, an antimalarial drug, inhibits dengue virus type 2 replication and infectivity. Antivir. Res..

[10] Delvecchio R., Higa L.M., Pezzuto P., Valadao A.L., Garcez P.P., Monteiro F.L., Loiola E.C., Dias A.A., Silva F.J., Aliota M.T. (2016). Chloroquine, an endocytosis blocking agent, inhibits Zika virus infection in different cell models. Viruses.

[11] Chatre C., Roubille F., Vernhet H., Jorgensen C., Pers Y.M. (2018). Cardiac complications attributed to chloroquine and hydroxychloroquine: A systematic review of the literature. Drug Saf..

[12] Manuja A., Kumar B., Chhabra D. (2022). Chloroquine Chaos and COVID-19: Smart delivery perspectives through pH sensitive polymers/micelles and ZnO nanoparticles. Arabian J. Chem..

[13] Manuja A., Kumar B., Riyesh T., Talluri T.R., Tripathi B.N. (2020). Microwave assisted fast fabrication of zinc/iron oxides based polymeric nanocomposites and evaluation on equine fibroblasts. Int. J. Biol. Macromol..

[14] Raguvaran R., Manuja A., Manuja B.K., Riyesh T., Singh S., Kesavan M., Dimri U. (2017). Sodium alginate and gum acacia hydrogels of zinc oxide nanoparticles reduce hemolytic and oxidative stress inflicted by zinc oxide nanoparticles on mammalian cells. Int. J. biol. Macromol..

[15] Manuja A., Raguvaran R., Kumar B., Kalia A., Tripathi B.N. (2020). Accelerated healing of full thickness excised skin wound in rabbits using single application of alginate/acacia based nanocomposites of ZnO nanoparticles. Int. J. Biol. Macromol..

[16] https://www.aatbio.com/tools/ic50-calculator-v1theequation.

[17] http://www.graphpad.com/quickcalcs/ttest1.cfm.

[18] Manuja A., Kumar B., Kumar R., Chhabra D., Ghosh M., Manuja M., Brar B., Pal Y., Tripathi B.N., Prasad M. (2021). Metal/metal oxide nanoparticles: Toxicity concerns associated with their physical state and remediation for biomedical applications. Toxicol. Rep..

[19] Neuman B.W., Kiss G., Kunding A.H., Bhella D., Baksh M.F., Connelly S., Droese B., Klaus J.P., Makino S., Sawicki S.G. (2011). A structural analysis of M protein in coronavirus assembly and morphology. J. Struct. Biol..

[20] Nal B., Chan C., Kien F., Siu L., Tse J., Chu K., Kam J., Staropoli I., Crescenzo-Chaigne B., Escriou N. (2005). Differential maturation and subcellular localization of severe acute respiratory syndrome coronavirus surface proteins S, M and E. J. Gen. Virol..

[21] Arndt A.L., Larson B.J., Hogue B.G. (2010). A conserved domain in the coronavirus membrane protein tail is important for virus assembly. J. Virol..

[22] Wu A., Peng Y., Huang B., Ding X., Wang X., Niu P., Meng J., Zhu Z., Zhang Z., Wang J. (2020). Genome composition and divergence of the novel coronavirus (2019-nCoV) originating in China. Cell Host Microbe..

[23] Solomon V.R., Lee H. (2009). Chloroquine and its analogs: A new promise of an old drug for effective and safe cancer therapies. Eur. J. Pharmacol..

[24] Mizushima N., Yoshimori T., Levine B. (2010). Methods in mammalian autophagy research. Cell.

[25] Li M., Khambu B., Zhang H., Kang J.H., Chen X., Chen D., Vollmer L., Liu P.Q., Vogt A., Yin X.M. (2013). Suppression of lysosome function induces autophagy via a feedback down-regulation of MTOR complex 1 (MTORC1) activity. J. Biol. Chem..

